# *Chlamydia trachomatis* infection among female inmates at Briman prison in Saudi Arabia

**DOI:** 10.1186/1471-2458-14-267

**Published:** 2014-03-20

**Authors:** Wafa Fageeh, Sami Badawood, Hanin Al Thagafi, Muhammad Yasir, Esam Azhar, Suha Farraj, Mona Alomary, Moneerah Alsaeed, Soonham Yaghmoor, Taha Kumosani

**Affiliations:** 1 Obstetrics and Gynecology Department, Faculty of Medicine, King Abdulaziz University, Jeddah, Saudi Arabia; 2 Health Affairs Western Region, Ministry of Health, Jeddah, Saudi Arabia; 3 Special Infectious Agents Unit, King Fahd Medical Research Center, King Abdulaziz University, Jeddah, Saudi Arabia; 4 Medical Laboratory Technology Department, Faculty of Applied Medical Sciences, King Abdulaziz University, Jeddah, Saudi Arabia; 5 Biochemistry Department, Faculty of Science, King Abdulaziz University, Jeddah, Saudi Arabia

**Keywords:** *Chlamydia trachomatis*, Female inmates, Briman Prison, Saudi Arabia

## Abstract

**Background:**

*Chlamydia trachomatis* infection is the most common sexually transmitted infection (STI) in the western countries; its prevalence in the conservative Muslim population of Saudi Arabia is not known, but it is generally believed to be low. This study is the first to investigate the prevalence of and risk factors for *C. trachomatis* infection in the high-risk group of female inmates at Briman Prison in Jeddah.

**Methods:**

The inmates were interviewed using a pre-designed questionnaire, and their urine samples were tested for *C. trachomatis* infection by real-time PCR assay.

**Results:**

The overall prevalence of *C. trachomatis* infection was 8.7% in the study population. The ≤25 age group was predominantly affected, with an average prevalence of 16.6%. Two out of five (2/5, 40%) Yamani, (4/33 12.1%) Indonesian, (3/33, 9.1%) Somalian and (2/26, 7.7%) Ethiopian inmates were positive for infection*.* None of the Saudi inmates (0/14) were positive for infection. Among the studied variables, only age was significantly associated with the infection rate. The other variables (marital status, nationality, religion, employment status, education level, nature of the offense committed, knowledge about protection from STIs, and knowledge about condom use and the purpose of condom use) did not show a significant correlation with Chlamydia infection.

**Conclusions:**

The overall prevalence of *C. trachomatis* infection was within the range published by other reports in similar prison settings in developed countries. The results indicate the need for a countrywide screening and treatment program for all inmates at the time of entry into prison.

## Background

*Chlamydia trachomatis* is the most prevalent sexually transmitted pathogen worldwide, and affects women disproportionately [1-4]. *C. trachomatis* infection is mostly asymptomatic and has long-term serious health consequences in women who are not screened on time and given proper treatment. In particular, ectopic pregnancy, pelvic inflammatory disease, urethritis, infertility and cervical carcinoma are long-term complications associated with chronic *C. trachomatis* infection [5-7]. The *C. trachomatis* infection rate in the general population in the West is 1–10% in both genders [2,8,9].

The risk of acquiring *C. trachomatis* is associated with several socio-demographic and behavioral factors [10]. Particularly, incarcerated persons are at a high risk for sexually transmitted infections (STIs). Inmates report multiple behaviors which increase the risk of STIs such as *C. trachomatis*, including sex with multiple partners, unprotected sex and inconsistent condom use, and substance use disorders [10-12]. Commercial sex work, which is frequent among incarcerated women, has also been associated with STIs [13,14]. Importantly, those infected with *C. trachomatis* may be at an increased risk of acquisition and transmission of HIV [15,16]. The surveillance program of Centers for Disease Control and Prevention (CDC, http://www.cdc.gov/std/) and other studies have reported a 7–21% prevalence of *C. trachomatis* infection among incarcerated females [17].

A very limited number of studies have been published about the prevalence of *C. trachomatis* in Islamic countries [18,19], and there is no report about *C. trachomatis* prevalence in the general population of Saudi Arabia. The general perception is that the prevalence of STI infections is low in Muslim societies due to prohibition of non-marital sex and sexual promiscuity. There has been no national study and there is no surveillance program in the Saudi Arabia to monitor the prevalence of *C. trachomatis* infection. The aim of this study is to initiate a program for monitoring the prevalence and spread of *C. trachomatis* infection in the country.

In this study, we evaluated the prevalence of *C. trachomatis* infection in incarcerated women. We hypothesized that this group of marginalized women would be likely to have a relatively high prevalence of STIs. This population also interacts with the general population after release, so diagnosis and treatment could affect the burden of disease in the general population [20]. The results of this study could significantly influence the development of appropriate policies regarding screening and treatment in prisons [21].

## Methods

### Study design

A cross-sectional study design was utilized in the present study to estimate the prevalence of *C. trachomatis* infection in the high-risk group of female prisoners. The study was conducted at Briman Prison in Jeddah. Jeddah is a metropolitan seaport city of Saudi Arabia that has the major population of expatriate workers, and Briman Prison is one of the biggest in the country. The female inmates comprise of local Saudi and expatriate workers from different countries.

### Ethical considerations

The Institutional Review Board of the Ministry of Health Western Province of Saudi Arabia permitted the STI screening and medical care program for inmates who were diagnosed with the infection. Written consent was obtained from each participant, and the study aims were explained verbally to those inmates with poor reading skills. Screening was conducted confidentially.

### Participants and procedure

Once participants provided their informed consent, an interview was conducted with each participant in a private room to ensure confidentiality. Data on sociodemographic characteristics, sexual behaviors (marital status, condom use, etc.), education, religion, employment status, and the nature of offense were included to identify risk factors for *C. trachomatis* infection in this population. We arranged for the medical supervisors of the prison to inform inmates who tested positive, and suggestions for appropriate treatment were given according to the recommendations of the CDC (http://www.cdc.gov/std/treatment/2010/chlamydial-infections.htm).

### Chlamydia testing

Urine samples were collected from participants in sterile containers. Samples were transported to the Special Infectious Agents Unit (SIAU) of King Fahd Medical Research Center. Real-time PCR analysis was used to detect *C. trachomatis* infection, using a modified procedure of Jaton et al. (2006) [22]. Briefly, urine samples were centrifuged at 14000 rpm for 20 min, and DNA was extracted from the pellet using the QIAamp DNA Mini Kit (Qiagen), following the manufacturer’s instructions. Real-time PCR amplification was performed using the primer pair Ctr_F (5′-CATGAAAACTCGTTCCGAAATAGAA-3′) and Ctr_R (5′-TCAGAGCTTTACCTAAC AACGCATA-3′), which amplifies a 71-bp DNA segment of *C. trachomatis*[22]. The oligonucleotides were prepared by MOLBIOL (Germany), and the cycles were performed using Rotor-Gene Q (Qiagen).

### Statistical analysis

Univariate analysis was used to determine the relationship between *C. trachomatis* infection (response variable) and individual explanatory variables (age, nationality, religion, marital status, nature of offense, level of education, profession, knowledge about protection against STIs, knowledge about whether the use of condom protects against STIs and prevents pregnancy). The independent-sample *t*-test was applied to ascertain the relationship between Chlamydia infection and age. Fischer’s exact test and the chi square test were applied to ascertain the relationship between the response variable and explanatory variables. A significant threshold was defined as a *P* value of <0.05. All statistical analyses were performed using SPSS version 20.

## Results

### Participant characteristics

A total of 211 female inmates of Briman prison participated in this study. Among them, 205 answered the questionnaire, and six returned incomplete questionnaires and were excluded from the analyses. The age range was 17–60 years, and the mean (±SD) age was 33.2 ± 9.3 years. In the study population, 7.8% of the inmates were of Saudi Arabian nationality. The majority of them, i.e. 90.7%, were expatriates; 32.2% were Indonesian; 2.9%, Yamani; 42.9% Africans (Ethiopia, Somalia, Chad, Sudan, Nigeria, Eretria and Egypt) and 14.2% other nationalities. Among the participants, 58.5% were convicted for prostitution, 10.2% for illegal immigrantion, 6.8% were convicted for wine trading, and the rest were convicted for forgery, theft, begging, etc. A majority of the participants were either married (39.0%) or divorced (46.3%). With regard to education, 34.1% were uneducated and only 4.9% were college graduates. With regard to knowledge about STIs, 52.2% of the incarcerated females were aware about protection from STIs; only 4.9% used condoms for protection from STIs; and 15.1% used condoms to prevent pregnancy. Moreover, more than 50% were not in favor of screening for STIs before marriage.

### Non-screened participants

64 participants opted out of screening. Background variables were almost the same among the tested and non-tested participants for Chlamydia infection. With regards to age, 78.1% (50/64) of the non-tested inmates for Chlamydia were above 25 years. Among the tested participant, 74.8% inmates were above 25 years of age. The majority of non-tested inmates were expatriates similar to the tested participants including Indonesian (33/64, 51.6%), Yemeni (1/64, 1.6%), African countries (Ethiopia, Somalia, Chad, Sudan, Nigeria, Eretria and Egypt) (17/64, 26.6%) and other nationalities (12/64, 18.8%).

Consistent with the participants tested for Chlamydia, majority of the non-tested participants were convicted for prostitution (49/64, 76.6%), illegal immigration (3/64, 4.7%), forgery (3/64, 4.7%), murder (2/64, 3.1%), drugs (1/64, 1.6%), wine trading (1/64, 1.6%) and others were convicted with theft and financial matters etc. Majority of the non-tested participants were either married (23/64, 35.9%) or divorced (29/64, 46.9%). In-contrast to tested participant, 42.2% (27/64) non-tested participant were educated to secondary school level, 18.8% (12/64) were uneducated and only 3.1% (2/64) were college graduates. With regard to knowledge about STIs, 42.2% (27/64) of the non-tested incarcerated females thought that they were aware about protection from STIs; only 9.4% (6/64) used condoms for protection from STIs; and 14.1% (9/64) used condoms to prevent pregnancy. Moreover, more than 42.2% (27/64) were not in favor of screening for STIs before marriage.

### Chlamydia infection and association with risk factors

Among the participants, 64 did not provide a urine sample. Therefore, we were able to test only 149 women for *C. trachomatis* infection. Thirteen were positive for *C. trachomatis* infection, which reflected an overall prevalence rate of 8.7% in the female inmates of Briman Prison. However among the 149 tested women, 6 participants including one positive female were excluded from the rest of analyses because they returned an incompletely filled questionnaire. With regard to age, 74.8% of the inmates who participated were above 25 years. Among the participants aged ≤25 years, 25.2% were sexually active, and the prevalence of *C. trachomatis* infection in this age group was 16.6% (Table 1). However, only 5.6% of the inmates aged above 25 years were positive for *C. trachomatis* infection. Among the participants who were tested for Chlamydia, 9.7% were Saudi Arabian; 23.1%, Indonesian; 3.5%, Yamani; 54.4% Africans (Somalia, Ethiopia, Chad, Sudan, Nigeria, Eretria and Egypt) and 9.8% other nationalities. None of the Saudi Arabian inmates were positive for *C. trachomatis* infection. Among the five Yamani inmates, two were positive for infection (2/5, 40%). In addition, the urine samples of (4/33, 12.1%) Indonesian, (3/33, 9.1%) Somalian and (2/26, 7.7%) Ethiopian inmates were positive for *C. trachomatis* infection. Nine of the twelve *C. trachomatis*-positive inmates were involved in prostitution or adultery; the remaining three *C. trachomatis*-positive inmates were convicted for wine trading and forgery. The infected participants were either married (10.5%), divorced (3.0%) or widowed (25.0%), and 12.5% of single female participants were positive for *C. trachomatis* infection. The infection rate was 8.2% and 11.8% in uneducated and primary-level educated inmates respectively. The infection rate was 10.3% among participants who were not aware of protection from STIs. The inmates who reported using a condom were free from infection. No significant difference was observed in the infection rate among women who preferred to use condoms only to prevent pregnancy compare to non-condoms user to prevent pregnancy. Only age was significantly associated with the *C. trachomatis* infection rate, as the prevalence was observed to decrease with age. The other variables did not show any significant association with Chlamydia infection.

**Table 1:** **Potential risk factors associated with *C. trachomatis *** **infection in the inmates of Briman Prison, Jeddah**

**Risk factors**	**% Participants**	**% Chlamydia-positive**	***p *** **value**
	**(N = 143)**	**(n = 12)**	
**Age**			
≤25 y	25.2	16.6	0.03
≥25 y	74.8	5.6	
**Marital Status**			
Single	5.6	12.5	0.06
Married	39.9	10.5	
Divorced	46.2	3.0	
Widow	8.4	25.0	
**Nationality**			
Saudi	9.7	0	0.5
Indonesian	23.1	12.1	
Somalian	23.1	9.1	
Ethiopian	18.1	7.7	
Yamani	3.5	40	
Unknown nationality	9.8	7.1	
**Religion**			
Muslim	94.4	8.9	0.64
Christian	3.5	0	
**Education**			
Uneducated	42.7	8.2	0.87
Primary	23.8	11.8	
Secondary	27.3	7.7	
Graduation	5.6	0	
**Employment status**			
Employed	72.0	8.7	0.9
Unemployed	23.1	6.1	
**Offense**			
Prostitution	50.3	12.5	0.7
Wine trading	9.1	15.4	
Forgery	4.9	14.3	
**Knowledge about protection from STIs**			
Yes	57.3	7.3	0.53
No	40.6	10.3	
**Knowledge about condom use**			
Yes	21.0	10.0	0.85
No	30.8	11.4	
**Condom use for protection from STIs**			
Yes	2.1	0	>0.99
No	96.5	8.6	
**Condom use for prevention of pregnancy**			
Yes	14.7	9.5	0.69
No	77.6	8.1	

## Discussion

Saudi Arabia has a conservative Muslim community, and information about STIs in Islamic countries is notably limited. This analysis of *C. trachomatis* infection among female inmates of Briman Prison is the first such study on the epidemiology of bacterial STIs in a prison population within the Saudi Arabia.

We found an 8.7% prevalence rate among the female inmates, which is consistent with the CDC (2005) Chlamydia surveillance report, according to which the prevalence of *C. trachomatis* infection is 8.9% among female inmates, and findings from other studies performed in similar settings to screen this high-risk group [4,23]. However, the prevalence rate reported by studies in western countries is varied between 3.9% and 21% [4,24]. Recently performed studies in antenatal clinics of the Saudi Arabia found that 10.5% of pregnant women and 7.8% of infertile Saudi women were positive for *C. trachomatis* infection [18,25].

In this study, the age of inmates was positively correlated with the infection rate, which was higher at 16.6% in inmates who were ≤25 years. A number of other studies have also reported high-risk behavior in young sexually active females, and young age is considered as a primary risk factor for STDs [17]. Moreover, the *C. trachomatis* infection rate in imprisoned adolescent females are reported to be as high as 24.7% [17,26]. Therefore, the prevalence of STIs in incarcerated adolescent females in the country is an important health concern, and it is important that all imprisoned women be screened for STIs [27-29].

In contrast with previous literature, prostitution and other factors associated with STIs including illiteracy, alcohol consumption, condom use and income status were not found to be significant risk factors in this study [30,31]. This may be because of the relatively small sample size and low prevalence of Chlamydia infection in our study population. Overall knowledge about protection from STIs was low in the studied participants. Consistent with studies from the Western world [32], 21.0% of incarcerated women were aware of condom use but only 2.1% actually used condoms for protection from STIs.

Apart from the small sample size, another limitation was that 31.8% of the participants either did not provide samples for testing or did not fill out the questionnaire. The reasons for refusal were not collected, but it is possible that this was because the majority of the inmates were expatriates and uneducated. They may have not understood the aims of study clearly or they may have had a general attitude of mistrust towards how the study information might be used.

Despite its shortcomings, this study provides a strong foundation for the initiation of screening programs for STDs in this high-risk inmate population in Saudi Arabia. Notably, since a majority of the prisoners are released back into their home communities, their health status is closely linked to that of the local population. Thus, comprehensive identification and treatment of STDs in prisons can limit the spread of infections from this high-risk population after their release [33].

## Conclusions

In conclusion, the overall prevalence of *C. trachomatis* infection (8.7%) in the female inmates at Briman prison is comparable to the infection rate reported in prison settings in developed countries. Age was significantly associated with infection rate. Future programs in this population should include regular screening for STDs, treatment, and spread of education and knowledge about protection from STDs. The programs established should also take into consideration the conservative nature of the society and be customized to the individual needs of inmates.

## Competing interests

There is no conflict of interest for this study.

## Authors’ contributions

WMKF, conceived and designed the study, supervised both samples and data collection and participated in writing the manuscript, data analysis and interpretation. SB, organized the project. HALT collected samples and data. MY performed the data analysis and drafted the manuscript. EA, supervised laboratory testing. SF, MA, MA and SY carried out laboratory sample testing. SY, assisted in project management as well. KT was in charge of the financial management and organization of the project. All authors read and approved the final manuscript.

## Pre-publication history

The pre-publication history for this paper can be accessed here:

http://www.biomedcentral.com/1471-2458/14/267/prepub
